# Appraisal of the Accuracy and Reliability of Cone-Beam Computed Tomography and Three-Dimensional Printing for Volumetric Mandibular Condyle Measurements of a Human Condyle

**DOI:** 10.7759/cureus.46746

**Published:** 2023-10-09

**Authors:** Ahmed M Elrawdy, Mohamed E Amer, Ahmed K Algariah, Mohamed H Eid, Abdelghafar M Abu-Elsaoud, Mohamed M Ghoneim

**Affiliations:** 1 Department of Oral Radiology, Suez Canal University, Faculty of Dentistry, Ismailia, EGY; 2 Department of Orthodontics, Zagazig University, Faculty of Dentistry, Zagazig, EGY; 3 Department of Orthodontics, Sinai University, Faculty of Dentistry, Ismailia, EGY; 4 Department of Oral and Maxillofacial Surgery, Suez Canal University, Faculty of Dentistry, Ismailia, EGY; 5 Department of Biology, College of Science, Imam Mohammad Ibn Saud Islamic University, Riyadh, SAU; 6 Faculty of Science, Suez Canal University, Ismailia, EGY; 7 Department of Oral and Maxillofacial Surgery, Sinai University, Faculty of Dentistry, El-Arish, EGY

**Keywords:** human condyle, pycnometer, condyle, 3d printing, volumetric measures

## Abstract

Background

This study aims to evaluate the accuracy of volumetric measurements of three-dimensional (3D)-printed human condyles from cone-beam computed tomography (CBCT) in comparison to physical condyles using a water displacement test.

Methodology

A sample of 22 dry condyles was separated from the mandibular body by disc, mounted on a base made of casting wax, and scanned using the SCANORA (Scanora 3DX, Soredex, Finland) CBCT scanner. Subsequently, the projection data were reconstructed with the machine-dedicated OnDemand 3D (Cybermed Co., Seoul, Korea). The Standard Tessellation Language file was prepared for 3D printing using chitubox slicing software v1.9.1. Frozen water-washable gray resin was used for 3D printing. All condyles were printed using the same parameters and the same resin. The volumetric measurements were then performed using a customized modified pycnometer based on water volume and weight displacement. Volumetric measures were performed for both the physical human condyles and the 3D-printed replicas and the measurements were then compared.

Results

The volume of dry condyles using the water displacement method showed an average (±SD) of 1.925 ± 0.40 cm^3^. However, the volume of 3D-printed replicas using the water displacement method showed an average (±SD) of 2.109 ± 0.40 cm^3^. The differences in measurements were insignificant (p > 0.05), as revealed by an independent t-test.

Conclusions

Highly precise, accurate, and reliable CBCT for volumetric mandibular condyle was applied for measurements of a human condyle and 3D-printed replica. The modified pycnometer for volumetric measurements presented an excellent volumetric measure based on a simple water displacement device. The tested modified pycnometer can be applied in volumetric measurements in both 3D-printed and mandibular condyle. For best accuracy, the highest scanning resolution possible should be used. As it directly handles irregularly shaped solid objects in a non-destructive manner with a high level of precision and reliability, this 3D scanning approach may be seen as a superior alternative to the current measurement methods.

## Introduction

Cone-beam computed tomography (CBCT) is an accurate and reliable method for measuring craniofacial structures [1,2]. Several studies have assessed its accuracy and reliability by scanning dry skulls to compare linear and volumetric measurements taken from physical structures and CBCT images [3,4].

Kayipmaz et al. [5] and Sezgin et al. [6] demonstrated the accuracy of CBCT for measuring volumes. Both studies applied Cavalieri’s principle to CBCT images and compared the results with physical volume calculations based on the Archimedean principle [5,6].

As the morphology and dimensions of the mandibular condyles play an important role in temporomandibular disorders [7], facial asymmetries [8], and certain malocclusions [9], their assessment is of the utmost importance in diagnosis. For this reason, an accurate and precise measurement method is crucial.

The use of three-dimensional (3D) printing in maxillofacial surgery started 30 years ago [10] but remained, until recently, in the hands of the industry. For about the past 10 years of general use, the availability of low-cost 3D printers has revived surgeons’ interest in this technology. Applications appear to have become broader, going from simple anatomic models to patient-specific implants, including cutting or drilling guides [11,12]

3D printing of anatomical structures with reliable and accurate volumetric measurements can facilitate the envisioning of deformities and minor asymmetries and enable the clinician to visualize the treatment options with sufficient clarity [11,13]. While proper volume measurement requires 3D pictures, the flatbed scanner only creates two-dimensional images. These imaging techniques have the advantages of repeatability, non-destructive sample handling, automated computations, computation speed, accuracy, and process automation viability [14].

Our study aims to evaluate the accuracy and reliability of such modified and customized volumetric measurements using a pycnometer of the mandibular condyle using a gold standard method of water displacement test for both the physical condyles and the 3D-printed replicas.

## Materials and methods

Sample size calculations

The current research was performed to assess the reliability between observers and compare the CBCT and physical methods for the volume determination of both dry condyles and 3D-print condyles. A minimum total sample size of 22 samples was sufficient to detect the effect size of 0.29, at a power (1-β = 0.90) of 90% at a significance probability level of p <0.05, and a partial eta squared of 0.08. According to sample size calculations, 24 samples were needed. The sample size was calculated according to G*power software version 3.1.9.6 [15-17].

The current research was performed after obtaining approval from the Research Ethics Committee Faculty of Dentistry, Sinai University (approval number: OMS 1-03-022, 2022).

Mandibular condyle scanning

A total of 24 well-preserved dry human skulls, used for educational and research purposes, were obtained from the Department of Anatomy, Suez Canal University (Ismailia, Egypt). The mandibular condyles were separated from the mandibular body by a disc. Then, condyles were mounted on a base made of casting wax. The mandibular condyle was scanned by SCANORA CBCT scanner (Scanora 3DX, Soredex, Finland). The field of view (FOV) and exposure parameters were selected and fixed for all samples as follows: FOV of 80 × 165 mm, 90 kV, 10 mA, exposure time of 2.4 seconds, and standard resolution made with a voxel size of 0.350 mm using a flat panel detector. The vertical and horizontal light-positioning guides were used to position the condyle that was rested on the chin rest and adjusted as directed by the manufacturer. The projection data were reconstructed with the machine-dedicated OnDemand 3D (Cybermed.Co., Seoul, Korea.) software application.

Image analysis and CBCT volume calculation

Image analysis was performed using the built-in OnDemand 3D software application. For volumetric measurements, the acquired image data were transferred into the DICOM format, followed by segmentation to create the condyles out of a 3D volume. The volumetric assessment of the segmented condyle was automatically calculated when the Segmentation function was used.

**Figure 1:**
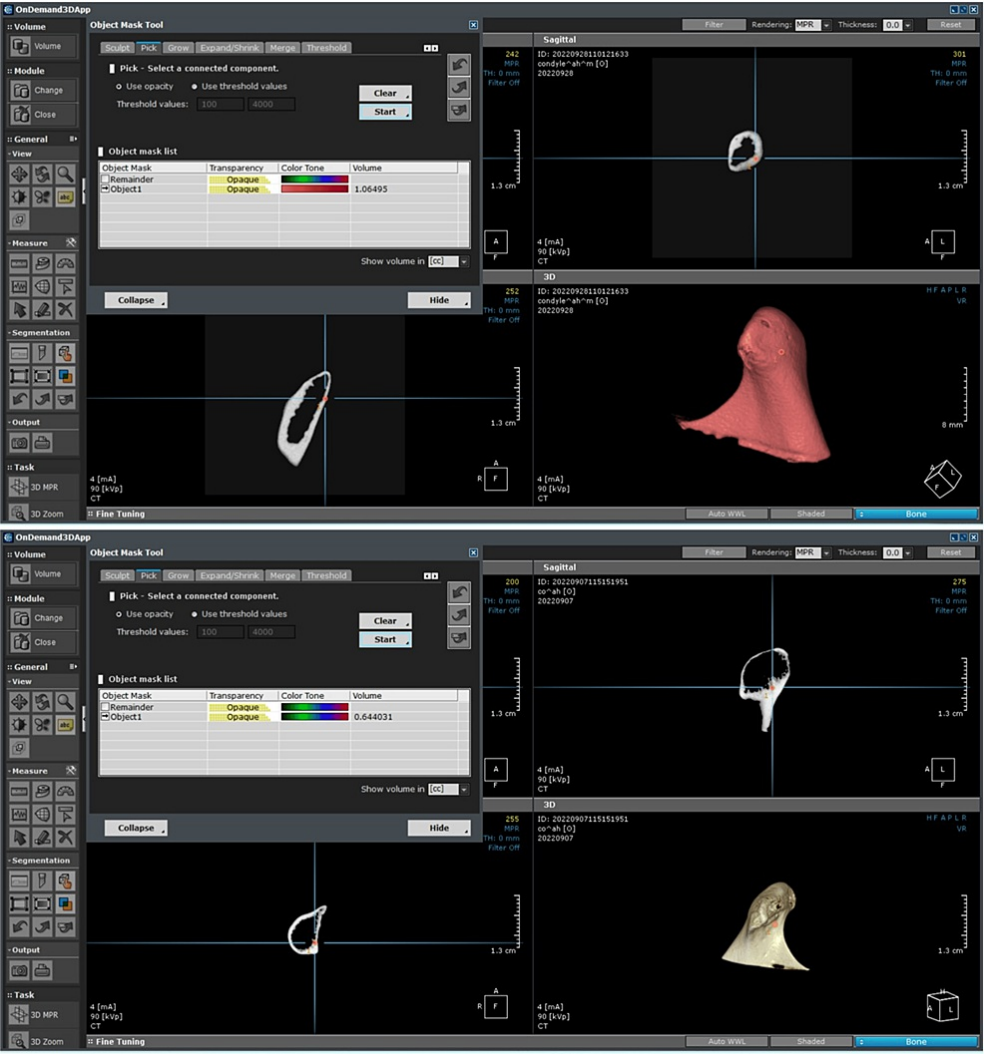
Image analysis performed using the built-in OnDemand 3D software. Image analysis using the built-in OnDemand 3D software application. For volumetric measurements, the acquired image data was transferred into the DICOM format. Then, segmentation was performed to create the condyles out of a 3D volume.

**Figure 2:**
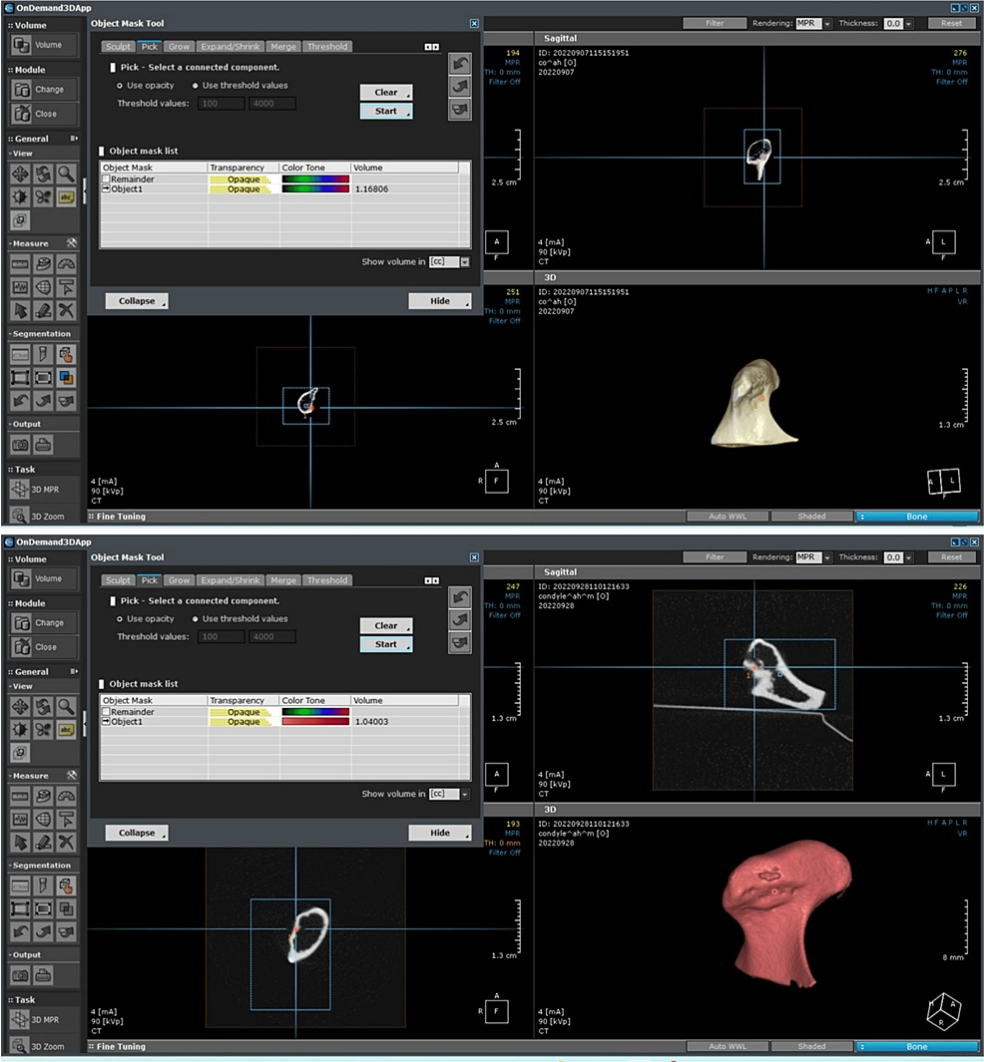
Image analysis performed using the built-in OnDemand 3D software.

**Figure 3:**
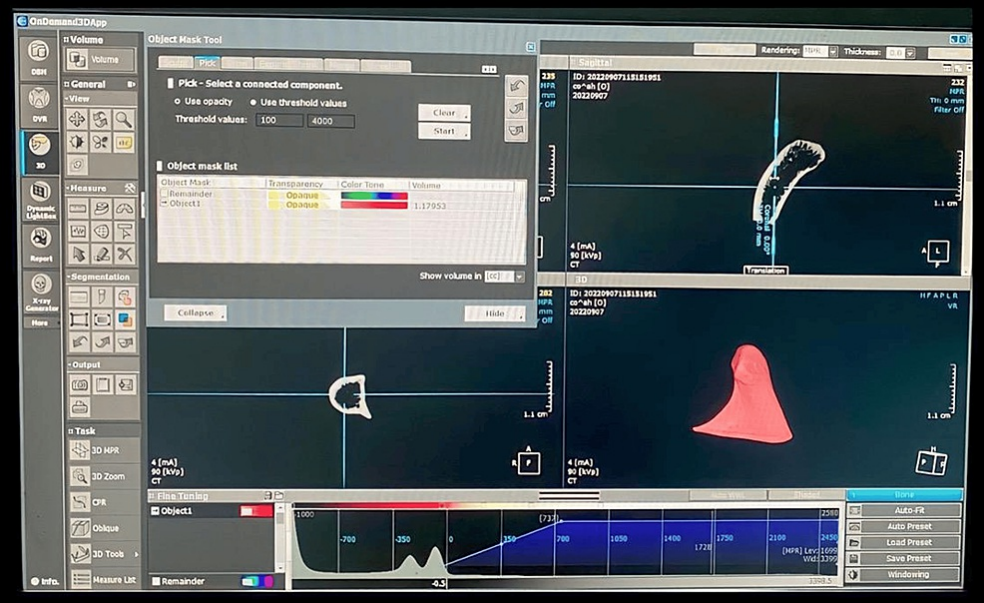
Image analysis performed using the built-in OnDemand 3D software.

Automatic segmentation was performed as follows: (1) the type of volume rendering options (bone) was first selected; (2) the Auto Preset tool was pressed to set the proper opacity and density values automatically for the selected rendering type; (3) the software automatically calculated the volume of the condyle already selected in cubic centimeters (cc) (Figures 1-3). Volumetric analysis was performed twice by each investigator (operator 1 and operator 2 with five- and fifteen years of experience in oral radiology, respectively) at a one-week interval.

Condyle 3D printing

**Figure 4:**
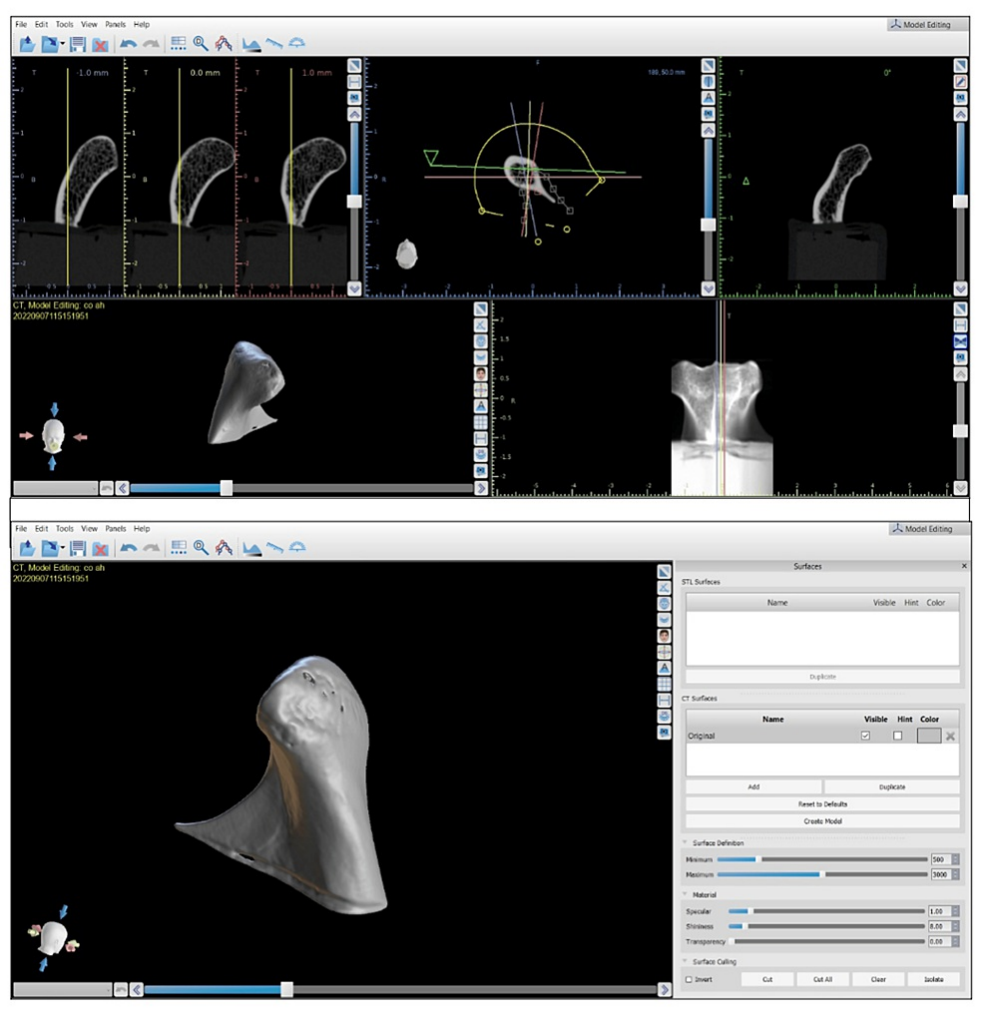
STL file format export of the condyle. The STL file was prepared for 3D printing using chitubox slicing software version 1.9.1.  Supporting bases were added to the condyle with a cone contact shape, contact diameter of 1 mm, and contact depth of 0.6 mm. Phrozen Sonic Mighty 4K LCD Resin 3D Printer was used for printing condyles.

To reproduce the exact dry condyle structure, condyles were 3D reconstructed through the CBCT rendering image function with the aid of blue-sky plan 4 software (Figure 4). The wax base was removed from the CBCT images using the software’s cutting tool. Then, the condyle was exported to the stereolithography Standard Tessellation Language (STL) file format (Figure 4).

The temporomandibular joint is the only joint in the stomatognathic system, and the shape of the mandibular condyle is one of the elements of the temporomandibular joint likely to be affected by functional pressure generated by occlusion and mandibular movements. Studies have reported that masticatory function and occlusal force are low in edentulous individuals. However, few thorough 3D studies have examined changes in the internal structures of mandibular condyles in edentulous individuals. Furthermore, few studies have quantitatively analyzed the shape of the various parts of the mandibular condyle.

The internal structures of mandibular condyles showed numerous plate-shaped trabeculae with continuity and regularity for dentulous jaws. However, with edentulous jaws, rod-shaped narrow trabeculae formed a mesh. Additionally, cortical bone thickness was thinner for edentulous jaws than for dentulous jaws in all regions. Internal structures of the mandibular condyle are influenced by reductions in functional pressure due to tooth loss, duration of denture usage, and the quality of denture fit, but denture usage and fit were unknown in this study, and the relationship with internal structures could not be ascertained.

Bone density and trabecular structure are important factors regulating the mechanical properties of bones. Trabecular number, trabecular thickness, and trabecular separation are indicators of bone structure strength. The study clarified that decreases in trabecular number and thickness reduce and reflect bone strength and that trabecular separation most sensitively reflects changes in bone metabolism.

When all teeth are lost, marked decreases occur in both trabecular volume in the articular tubercle and trabecular thickness. The reason for this was a decrease in functional pressure due to tooth loss, rather than age-related changes.

The internal structures of pediatric articular tubercles from the deciduous dentition period to the mixed dentition period, trabecular shapes change from rods to plates and trabecular connectivity increases, trabeculae inside the mandibular condyle and the upper trabeculae adjacent to the functional surface of jaw movements display high volume ratios and low anisotropy (i.e., the physical properties of materials differ in different directions), thus enabling adaptation to forces in any direction.

**Figure 5:**
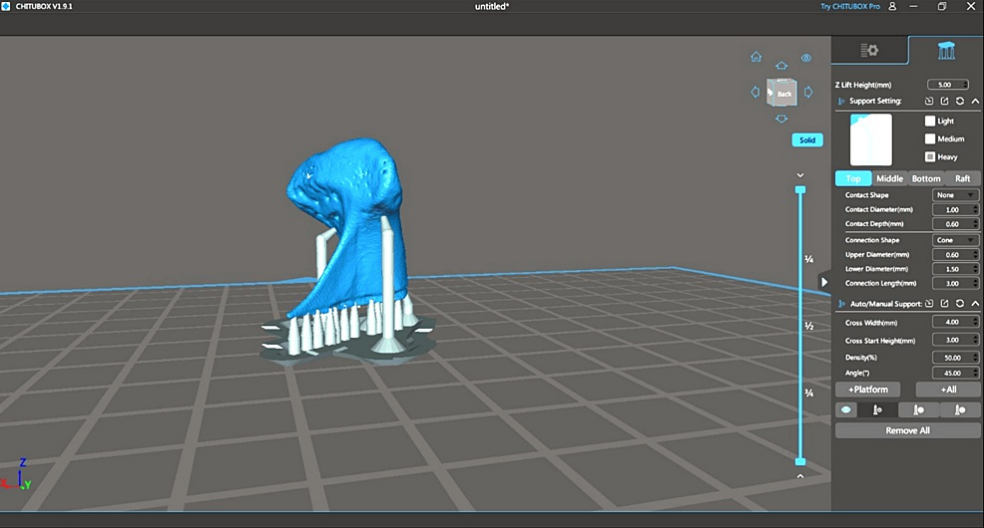
Base application on slicing software and 3D reconstruction of the condyle from the DICOM file. Phrozen water-washable grey resin was used for 3D printing. All the condyles were printed using the same parameters and the same resin.

The STL file was prepared for 3D printing using chitubox slicing software v1.9.1. Supporting bases were added to the condyle with a cone contact shape, contact diameter of 1 mm, and contact depth of 0.6 mm (Figure 5). Phrozen Sonic Mighty 4K LCD Resin 3D Printer was used for printing condyles. The parameters used for 3D printing were exposure time of 2.5 seconds, bottom exposure time of 40 seconds, lift distance of 8 mm, layer height of 0.05 mm, and lift speed of 60 mm/minute.

BlueSky Bio software employed for generating the 3D models from CBCT scans in our study does not provide explicit information about the generation algorithm, binarization threshold, linear interpolation, or polygon count reduction. It operates as a user-friendly tool that abstracts these technical details from the user, focusing on the efficient conversion of CBCT data into 3D models.

Furthermore, the primary objective of our study was to assess the accuracy of volumetric measurements of 3D-printed human condyles from CBCT in comparison to physical condyles using a water displacement test. As such, the specific technical parameters of the software’s internal processes are beyond the scope of our investigation.

**Figure 6:**
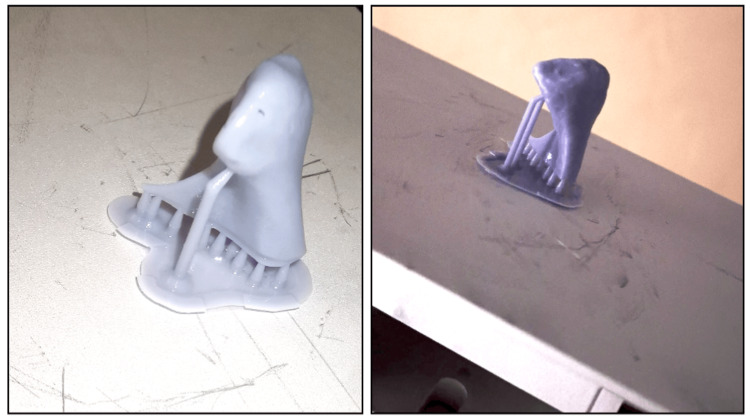
3D-printed condyles. Phrozen water-washable gray resin was used for 3D printing. All condyles were printed using the same parameters and the same resin.

Phrozen water-washable gray resin was used for 3D printing. All condyles were printed using the same parameters and the same resin (Figure 6). After the printing process, the printed condyle was separated from the build plate, and the supporting base was removed.

Water displacement technique

**Figure 7:**
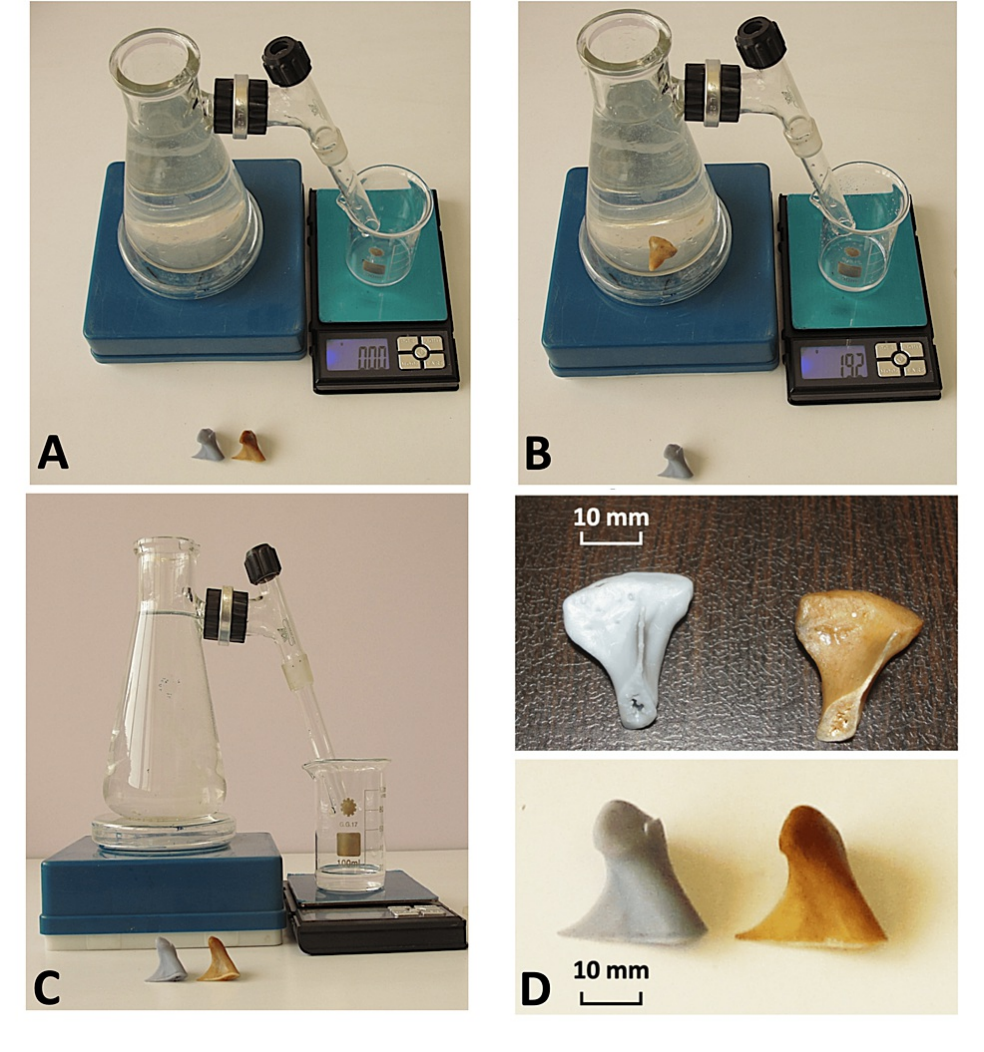
Modified water displacement device (pycnometer) for measuring condyle volume accurately using balancing and volume of water displaced. (A) water displacement device, (B) water displacement device settings with a condyle to measure the volume, (C) side view of water displacement volume, and (D) condyle view.

Volumetric measurements were performed on the dry condyles, where each condyle was immersed in a customized modified pycnometer, modified after Garcia-Sanz et al. [18] (Figure 7), which had been filled with water, and the volume and weight of the run-over water was determined (Figure 7). Weight and volume of run-over water were used to calculate the volumes of the condyles.

Statistical analysis

Data were collected, checked, revised, and organized in tables and figures using Microsoft Excel 2016 (Microsoft Corp., Redmond, WA, USA). Data were subjected to outliers’ detections and normality statistical tests to detect whether the data were parametric or non-parametric. Data were analyzed for descriptive statistics using both graphical and numerical descriptions. Interobserver agreement was performed using Cronbach’s alpha, Pearson’s correlation, linear regression, and interclass correlation at a 0.05 level. Statistical analysis was performed to check the agreement between observers and to compare the CBCT and physical methods for the volume determination of both dry condyles and 3D-printed condyles. A repeated-measure analysis of variance (ANOVA) test was used. ANOVA followed by Duncan multiple range tests (DMRTs) were used to compare the treatment groups or corresponding post-hoc tests were used for non-parametric data. SPSS version 28 (IBM Corp., Armonk, NY, USA) was used for data analysis [19]. Heatmap was generated using PAST statistical software version 4.11.

## Results

The results of the two observers are presented in Table 1. The average (±SD) volumes of dry condyles using CBCT and the physical methods were 2.278 ± 0.41 and 1.925 ± 0.40 cm^3^, respectively. However, the average (±SD) volumes of 3D-printed condyles using the CBCT and physical methods were 2.13 ± 0.40 and 2.109 ± 0.40 cm^3^. The interobserver differences within the four methods were non-significant (p > 0.05) as revealed by the paired-sample t-test (Table 1).

**Table 1:** Descriptive and confidence intervals (CI) of interobserver differences using the paired-sample t-test. ns: non-significant at p-values >0.05. CBCT: cone-beam computed tomography; CI: confidence interval; SD: standard deviation

Volume (cm^3^)		Observer 1		Observer 2		Difference		95% CI		Paired t-test	
		Mean	SD	Mean	SD	Mean	SD	Lower	Upper	t	P-value
Dry condyle	CBCT	2.278	0.41	2.270	0.43	0.007	0.15	-0.06	0.07	0.23	0.818 (ns)
	Physical	1.925	0.40	1.938	0.42	-0.013	0.12	-0.06	0.04	-0.5	0.612 (ns)
3D print	CBCT	2.130	0.40	2.143	0.42	-0.013	0.07	-0.04	0.02	-0.4	0.686 (ns)
	Physical	2.109	0.40	2.115	0.41	-0.006	0.07	-0.03	0.02	-0.4	0.681 (ns)

The interobserver reliability is presented in Table 2. Interobserver reliability within CBCT volume and physical volume of dry condyles showed a significant and high agreement and correlation (r = 0.94; Cronbach’s alpha = 0.97; p < 0.001 and r = 0.96; Cronbach’s alpha = 0.98; p < 0.001, respectively). Moreover, CBCT and physical volumes of 3D-printed condyles also showed a significantly high interobserver agreement (Table 2).

**Table 2:** Assessment of interobserver reliability using interclass correlation and Cronbach’s alpha. ***: highly significant at p-values <0.001. CBCT: cone-beam computed tomography; SD: standard deviation; ICC: interclass correlation

Volume (cm^3^)		Difference		Interobserver reliability			
		Mean	SD	r	Alpha	ICC	P-value
Dry condyle	CBCT	0.007	0.15	0.94	0.97	0.969	<0.001***
	Physical	-0.013	0.12	0.96	0.98	0.96	<0.001***
3D printed	CBCT	-0.013	0.07	0.99	0.99	0.986	<0.001***
	Physical	-0.006	0.07	0.99	0.99	0.986	<0.001***

**Figure 8:**
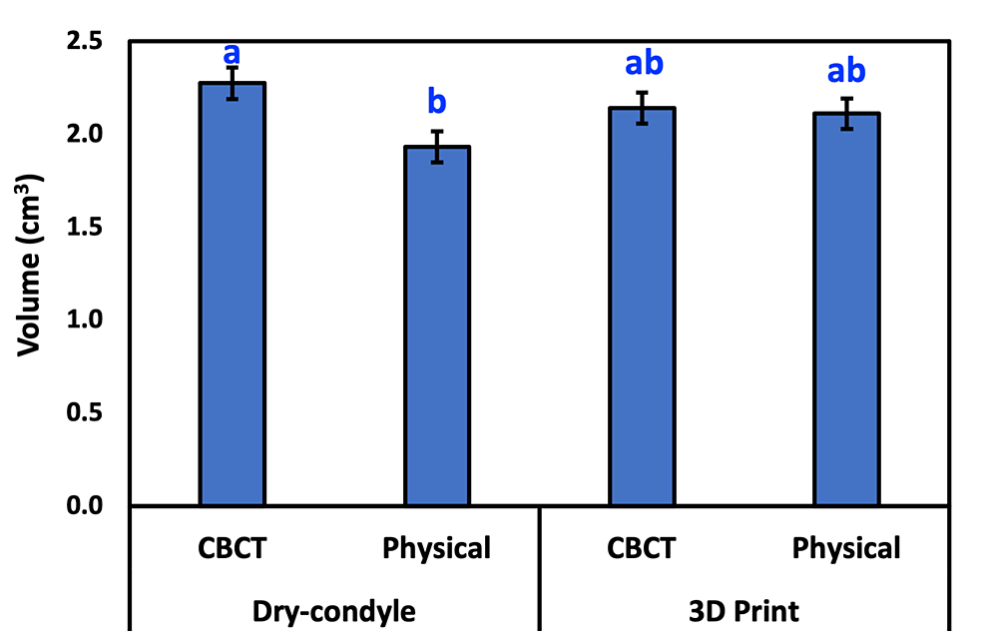
The estimated volume is either CBCT or physical of both dry condyle and 3D-print condyle. According to Duncan’s multiple range test, bars followed by different letters are significantly different. Bar represents the mean ± standard deviation (SD). CBCT: cone-beam computed tomography

**Figure 9:**
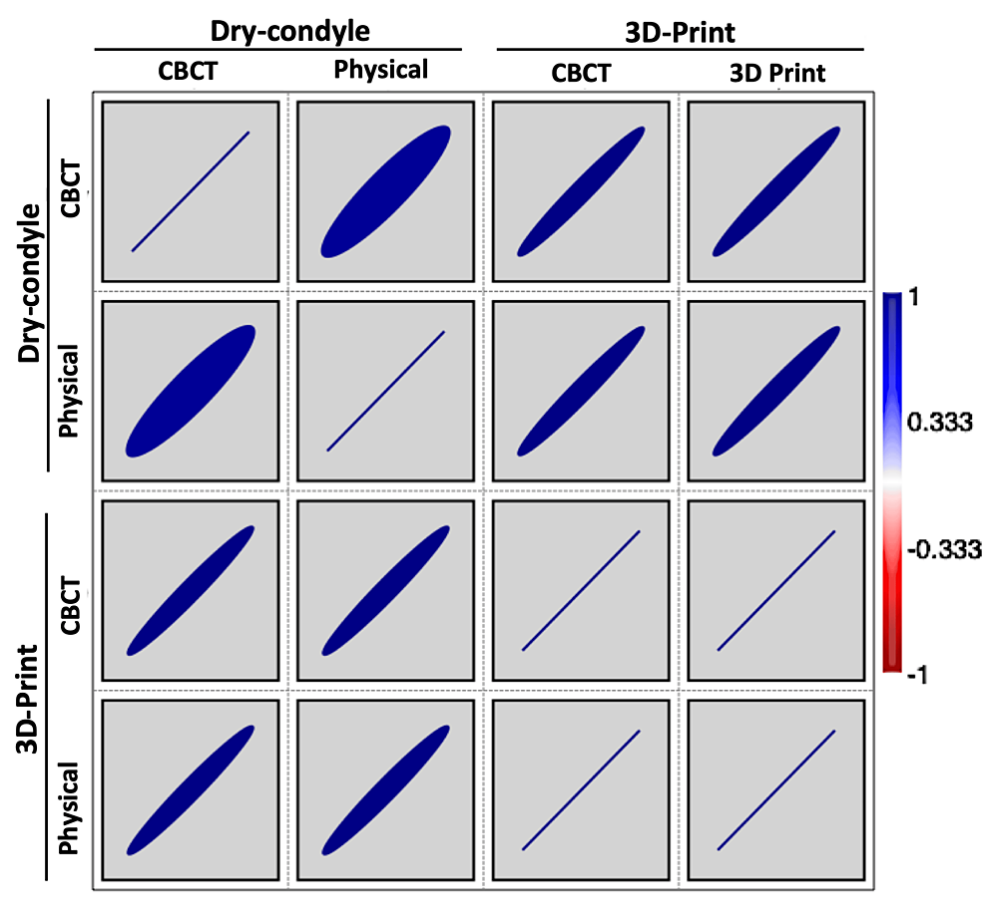
Blue-red heatmap showing the interrelationship between the dry condyle and 3D-printed condyle using both the CBCT and physical methods. CBCT: cone-beam computed tomography

**Figure 10:**
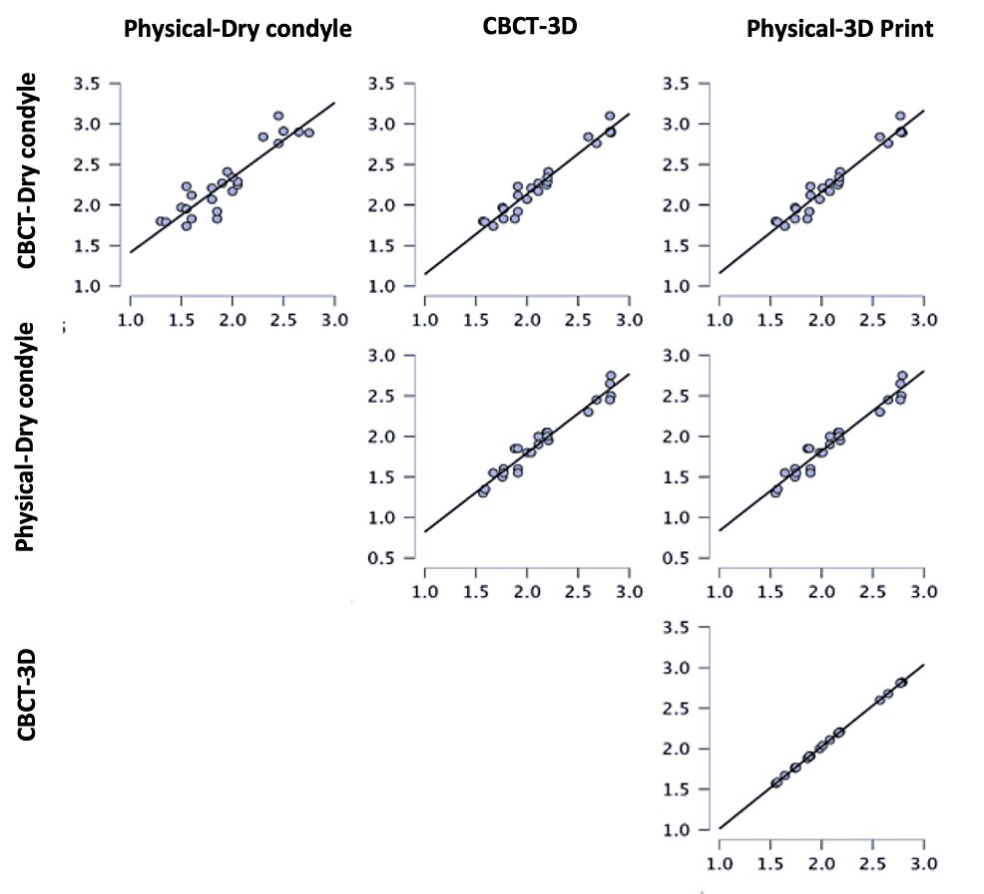
Regression trendlines showing the relationship between the dry condyle and 3D-printed condyle using both the CBCT and physical methods. CBCT: cone-beam computed tomography

The estimated volume of either the CBCT or physical methods of both dry condyles and 3D-printed condyles is presented in Table 3. The average (±SD) volume of dry condyles either by the CBCT and physical methods were 2.27 ± 0.416 and 1.93 ± 0.409, respectively. However, the average (±SD) volume of 3D-printed condyles either by the CBCT or physical methods were 2.14 ± 0.410 and 2.11 ± 0.404, respectively. The difference between the four groups was significant, as revealed by repeated-measures ANOVA (p = 0.042). According to DMRT, the means followed by different letters were significantly different (Figure 8). The relationship between the CBCT or physical methods of both dry condyles and 3D-printed condyles was very strong and significant (Table 4, Figures 9, 10).

**Table 3:** The estimated volume is either CBCT or physical of both dry condyle and 3D-printed condyle. *: significant at p-values <0.05. ANOVA: analysis of variance; CBCT: cone-beam computed tomography; CI: confidence interval; DMRT: Duncan multiple range test; SD: standard deviation; SEM: standard error of the mean

Group		Mean	DMRTs	SD	SEM	95% CI	
						Lower	Upper
Dry condyle	CBCT	2.274	a	0.416	0.085	2.099	2.450
	Physical	1.931	b	0.409	0.084	1.759	2.104
3D printed	CBCT	2.140	ab	0.410	0.084	1.967	2.313
	Physical	2.111	ab	0.404	0.083	1.941	2.282
ANOVA		0.042*					

**Table 4:** Pearson’s correlation between the dry condyle and 3D-print condyle using both the CBCT and physical methods. ***: highly significant at p-values <0.001. CBCT: cone-beam computed tomography

Group	Dry condyle		3D printed	
	CBCT dry	Physical dry	CBCT 3D	Physical 3D
CBCT dry		<0.001***	<0.001***	<0.001***
Physical dry	0.9089		<0.001***	<0.001***
CBCT 3D	0.9758	0.9752		<0.001***
Physical 3D	0.9763	0.9748	1.0000	

## Discussion

Maxillofacial surgery has evolved by 3D printing. The last 30 years have made it possible for surgeons to treat patients more precisely and individually [12,20]. In addition to producing prosthetic devices and patient-specific models for preoperative planning, 3D printing has been utilized to stabilize facial tissues immediately after surgery [2,12,13,21].

The use of 3D printing in maxillofacial surgery has enabled surgeons to plan more accurately and prepare more efficiently for procedures. With 3D printing, patient-specific models can be created that replicate the patient’s anatomy for preoperative planning [14]. This helps surgeons to better assess the situation before performing a procedure, as well as to plan more precisely and accurately for the best outcome. Additionally, 3D printing can be used to create implants that are customized to the patient, such as a mandibular implant or an orthognathic device [13]. These implants can be 3D printed in any shape, size, or material, allowing the patient to benefit from a more personalized treatment [11].

3D printing has also enabled surgeons to provide immediate postoperative stabilization of facial structures to create customized plates, pins, and screws [22]. This has allowed surgeons to better manage facial fractures and manage complications resulting from trauma or surgery [23].

3D printing is based on depicting the anatomical structure using an appropriate imagining technique stored in a standard format such as the DICOM format. A virtual 3D prototype can then be created by computer software in the STL format. Therefore, 3D printing can be achieved with layer-by-layer deposition of the chosen material [24].

CBCT has become an increasingly popular method for measuring craniofacial structures including condyles [2]. It has been proven to be a reliable and accurate method for measuring condyles due to its ability to provide information on the position, shape, and size of the condyles [25]. Furthermore, CBCT offers improved diagnostic accuracy when compared to traditional methods such as radiography, as it provides detailed 3D images [1,26,27]. Moreover, CBCT has been used to measure condyles in various orthodontic and orthopedic studies [28]. In orthodontic studies, CBCT has been used to measure the position of the condyles concerning the maxilla or mandible to determine the degree of malocclusion [29,30]. It has also been used to measure the size and shape of the condyles which can then be used to determine the severity of temporomandibular disorders [31]. In orthopedic studies, CBCT has been used to measure the position of the condyles to study their range of motion and the effects of treatment on their position [32].

Therefore, this study was conducted to verify the interobserver consistency and evaluate the relative merits of CBCT and physical measurement techniques for estimating the volume of dry condyles for 3D printing. Using the gold-standard technique of water displacement test, this study aimed to assess the accuracy and reliability of such volumetric measurements of the mandibular condyle for both the physical condyles and the 3D-printed replicas. For this study, the volume was stored in the DICOM format and the CBCT images were stored in the STL file format.

Mandibular condyles were the focus of this study because they are one of the most significant components of the temporomandibular complex. As a result, understanding the morphology of condyles is often helpful in gaining a better interpretation of certain disorders, and it is essential in making an accurate diagnosis. The findings of this study can be discussed under the following two main topics: (1) the interobserver differences within the volume of dry condyles using the CBCT and physical methods, on one hand, and the volume of the 3D-printed condyles using the CBCT and physical methods, on the other. The findings of this study showed that the four approaches were comparable with no statistical significance. (2) The interobserver reliability within CBCT volume and physical volume was high with a strong interobserver agreement, on one hand, and the interobserver agreement concerning the CBCT and physical volume of the 3D printing, on the other.

The craniofacial structures can be well studied using CBCT. Earlier in vivo studies investigated the anatomy of the condyle. Despite the presence of an association between the condyle volume and skeletal class [33], anatomical variability was high concerning the volume and the surface of the mandibular condyles [33]. However, these findings were countered by recent studies such as the study by Ceratti et al. (2022), who found no significant association between condylar volumes and skeletal class [34]. Moreover, the condylar volume could be accurately assessed in patients with temporomandibular involvement (rheumatic comorbidity) [35]. Applying CBCT to the cadaver, CBCT was proved to be accurate for condyle volume measurements. Similar results were obtained by investigating the accuracy of CBCT on nine mandibular condyles [36]. Furthermore, it was concluded that the condylar segmentation approach using CBCT was a reliable and accurate condylar volume evaluation tool [37]. Comparing semi-automatic segmentation of mandibular condyles using CBCT with manual segmentations showed no significant differences in the volumetric dimensions of the condylar models among the different four software [38].

Concurring with the most recent results, this study found that CBCT was highly accurate in detecting the volume of the condyles. The findings showed that there was no significant interobserver difference between the CBCT and physical approaches concerning the dry condylar volumes. In addition, the application of CBCT to detect the volume of 3D-printed replicas yielded comparable results to the physical methods.

Accumulating evidence from the literature showed that CBCT 3D reconstruction helps calculate the volume and surface of the mandibular condyle [39]. A systematic review of the literature concluded that the 3D volume of condyles detected by CBCT images showed a moderate amount of variance in reliability and accuracy depending on the applied technique [40]. Compared with different voxel sizes with digital intraoral imaging techniques, CBCT imaging diagnostic accuracy showed the highest sensitivity and specificity among the other imaging modalities [41]. For CBCT reliability, the calculated intra and interobserver error was calculated showing a high CBCT reliability [18]. Assessing linear measurements on multiplanar images, CBCT volume rendering was reliable concerning the dry mandible [42]. Using the Cavalieri principle, condyle volume measurements by CBCT were concluded to be a highly reliable approach [43]. Moreover, the use of CBCT and physical measurement to obtain the condylar volume showed excellent intraobserver reliability [37]. In addition, the use of CBCT for 3D printing showed excellent reliability for both intra and interobserver readings [38].

The Cavalieri principle was used in this study, and the results agreed with previous studies regarding the utilization of CBCT in determining the volume of the condyles. There were no significant interobserver differences. In addition, the application of CBCT for measuring the volume of 3D print showed a significantly high interobserver agreement.

Study limitations include taking into consideration various variables such as age, gender, and edentulism in CBCT evaluations. This study is subject to certain limitations that merit discussion. First, while our primary focus was on assessing the accuracy of volumetric measurements using a water displacement test, we acknowledge that variations in resin density, a parameter within the 3D printing process, were not the primary emphasis of our investigation. We assumed that resin material density would have minimal impact on our primary measurement of volume, grounded in the principle of Archimedes, where the volume of water displaced by an object is equal to the volume of the object itself. However, to provide a more comprehensive understanding of potential factors influencing our measurements, we intend to incorporate a dedicated section in the limitations of this paper, explicitly recognizing the potential influence of resin density.

Second, our investigation employed dried condyles as specimens for the assessment of volumetric measurements. While dried condyles are commonly utilized in anatomical research due to their preservation and accessibility, it is crucial to recognize that they may not fully replicate the properties and characteristics of living human condyles encountered in clinical scenarios. The dehydration process that dried condyles undergo can lead to changes in tissue properties, including tissue shrinkage and variations in bone density, in comparison to fresh or cadaveric human condyles. Therefore, the findings of this study should be interpreted within the context of dried condyles, with the understanding that clinical application may necessitate further validation on living or cadaveric specimens that more closely resemble clinical conditions.

## Conclusions

Within the limitations of this study and according to study findings, there was a high precision, accuracy, and reliability of CBCT for volumetric mandibular condyle measurements of a human condyle and 3D-printed condyle. The modified pycnometer for volumetric measurements presented an excellent volumetric measure based on a simple water displacement device. Therefore, the tested modified pycnometer could be of good application in volumetric measurements in both 3D-printed and mandibular condyle.
